# Gel and thermoluminescence dosimetry for dose verifications of a real anatomy simulated prostate conformal radiation treatment in the presence of metallic femoral prosthesis

**DOI:** 10.1002/acm2.13403

**Published:** 2021-08-26

**Authors:** Diana M. C. Rojas, Juliana F. Pavoni, Gustavo V. Arruda, Oswaldo Baffa

**Affiliations:** ^1^ Department of Physics Faculty of Philosophy, Sciences, and Letters University of São Paulo Ribeirão Preto Sao Paulo Brazil; ^2^ Department of Physics Faculty of Philosophy, Sciences and Letters, University of São Paulo Ribeirão Preto Sao Paulo Brazil; ^3^ Ribeirão Preto Medical School, University of São Paulo Ribeirão Preto Sao Paulo Brazil; ^4^ Department of Physics Faculty of Philosophy, Sciences and Letters, University of São Paulo Ribeirão Preto Sao Paulo Brazil

**Keywords:** femoral prosthesis, gel dosimetry, MAGIC‐f, prostate, thermoluminescent dosimeters

## Abstract

This study aims to verify the dose delivery of prostate radiotherapy treatments in an adult pelvic phantom with two metallic hip and femur prosthesis using a four‐field box technique. The prostate planned target volume (PTV) tridimensional (3D) dose distribution was evaluated using gel dosimetry, and thermoluminescent dosimeters (TLD) were used for point‐dose measurements outside it. Both results were compared to the treatment planning system (TPS) dose calculation without using heterogeneity corrections to evaluate the influence of the metal in the dose distribution. MAGIC‐f gel dosimeter (Methacrylic and Ascorbic acid in Gelatin Initiated by Copper with Formaldehyde) associated with magnetic resonance imaging was used. TLD were positioned at several points at the bone metal interface and the sacrum region. The comparison of the gel measured and the TPS calculated dose distributions were done using gamma analysis (3%/3 mm), and a pass rate of 93% was achieved. The TLD dose values at the bone‐metal interface showed variations from the planned dose. However, at the sacrum region, where the beams did not intercept the prosthesis, there was a good agreement between TPS planning and TLD measurements. Our results show how the combination of 3D dosimetry and measurements at specific points in the phantom allowed a comprehensive view of the dose distribution and identified that care must also be paid to regions outside the PTV.

## 1 INTRODUCTION

A considerable number of patients requiring radiotherapy for prostate cancer have one or two hip prostheses,^1^, ^2^, ^3^, ^4^, ^5^ which distorts the prostate region dose distribution due to the increased beam attenuation. It also increases the dose at the bone–metal interface due to scattered electrons. Thus, treatment planning for these patients becomes complicated. In addition to the dosimetric effect, the prosthesis presence also affects the delineation accuracy of the structures in the computed tomography (CT) images employed in the 3D planning process. When patients have prostheses, artifacts are generated in the CT images in the same treatment region, degrading the image quality, and limiting visualization of the target volume.^6^ Furthermore, the delineation accuracy of organs, such as the bladder, rectum, and hip joint, can also be affected. To avoid dosimetric uncertainties in the calculated dose distributions, it is recommended to use radiation beams in planning treatment for these patients which do not intersect the prosthesis. If applying direct radiation beams intersecting the metallic prosthesis, it is necessary to follow several procedures to evaluate the real influence of the metal in the target dose distribution, OAR, and peripheral tissue.^2^ However, the two previously described techniques present some difficulties.^2^ In the first option, the problem is that unconventional radiation beam directions result in dose distributions which may not limit the organ at risk (OAR) doses as expected and will require more efforts to generate acceptable plans. With the second option, the final plan target volume coverage is limited due to the prosthesis geometry in the beam and inaccuracies in the treatment planning system (TPS) algorithms for inhomogeneity correction. Also, other clinical issues related to the prosthesis fixation in the bone due to a possible increase in the dose received in their interface may be considered.

Sophisticated radiotherapy techniques allow us to solve undesirable dose enhancement problems given by materials with a high Z. Among these techniques, we can cite intensity‐modulated radiation therapy (IMRT), volumetric modulated arc therapy (VMAT), proton therapy, or stereotactic body radiosurgery. However, in some hospitals, there is neither permanent nor temporary access to such techniques, so that the patients with metallic implants are treated using the standard four‐field box technique and three‐dimensional conformal radiotherapy (3DCRT) technique. Although a complex dose distribution is delivered with 3DCRT, the dose gradients involved are smaller than those delivered with intensity‐modulated techniques. Therefore, the dose verification of 3D‐RT treatments to evaluate the metallic prosthesis effect are simpler than with IMRT treatments and can help to elucidate part of the problems associated with the planning dosimetry of beams crossing metallic prostheses.

Recently, many researchers have attempted to quantify the effect of different prosthetic devices in the treatment plan using a phantom containing the prosthesis and Monte Carlo methods or TPS^7^, ^8^, ^9^, ^10^, ^11^, ^12^ simulation. The most comprehensive document on this issue is the American Association of Physicists in Medicine (AAPM) Task Group Report 63.^2^ It shows how the dose distribution is affected when materials with a high Z are used in radiation oncology patients and discusses the perturbations in the dose distribution of titanium, cobalt‐chromium‐molybdenum (Co‐Cr‐Mo), and stainless steel prostheses. As a result, an attenuation in the radiation beam and a consequent reduction in the target volume dose was verified for these three materials. Warmington et al.^11^ showed the feasibility of using polymer gel dosimetry with tridimensional (3D) dose distribution measurement for evaluating this problem. They presented the 3D millimetric resolution dose measurement in the vicinity of the lead sheets inserted in the gel. This measurement was in agreement with the Monte Carlo simulation results. Polymeric gel dosimeters are composed of a gelatin matrix with monomers. Under irradiation, the produced water radiolysis radicals initiate a polymerization reaction which occurs as a function of the absorbed dose. The presence of polymer chains in the gelatin alters the mobility of water molecules, thereby allowing to quantify the absorbed doses by using R2 relaxometry in magnetic resonance images (MRI).

The use of metal implants in patients was verified to significantly affect the tumor dose, reducing the total dose received by the treated organ.^2^ The effect of the treatment in regions close to the high Z materials was also verified.^9^ However, no study has simultaneously evaluated the dose at the target volume or in the neighboring regions of the metallic implants, and therefore, the aim of this study is to do so. A four‐field box conformal treatment dose distribution planning was studied. The target volume doses were evaluated in 3D using MAGIC‐f (Methacrylic and Ascorbic acid in Gelatin Initiated by Copper with Formaldehyde) gel dosimeter.^13^, ^14^, ^15^, ^16^, ^17^, ^18^ At the same time, thermoluminescent dosimeters (TLD) monitored the treatments near the prosthesis and other sites in the vicinity of the treatment region. All the measurements used a pelvis phantom composed of acrylic walls filled with water and containing the pelvic region of an adult with two stainless steel femoral prostheses. The achieved results were compared with the TPS data without the use of heterogeneity corrections to evaluate the metal's real influence without uncertainties associated with the calculation algorithm.

## 2 MATERIALS AND METHODS

### 2.1 Phantom

**FIGURE 1:**
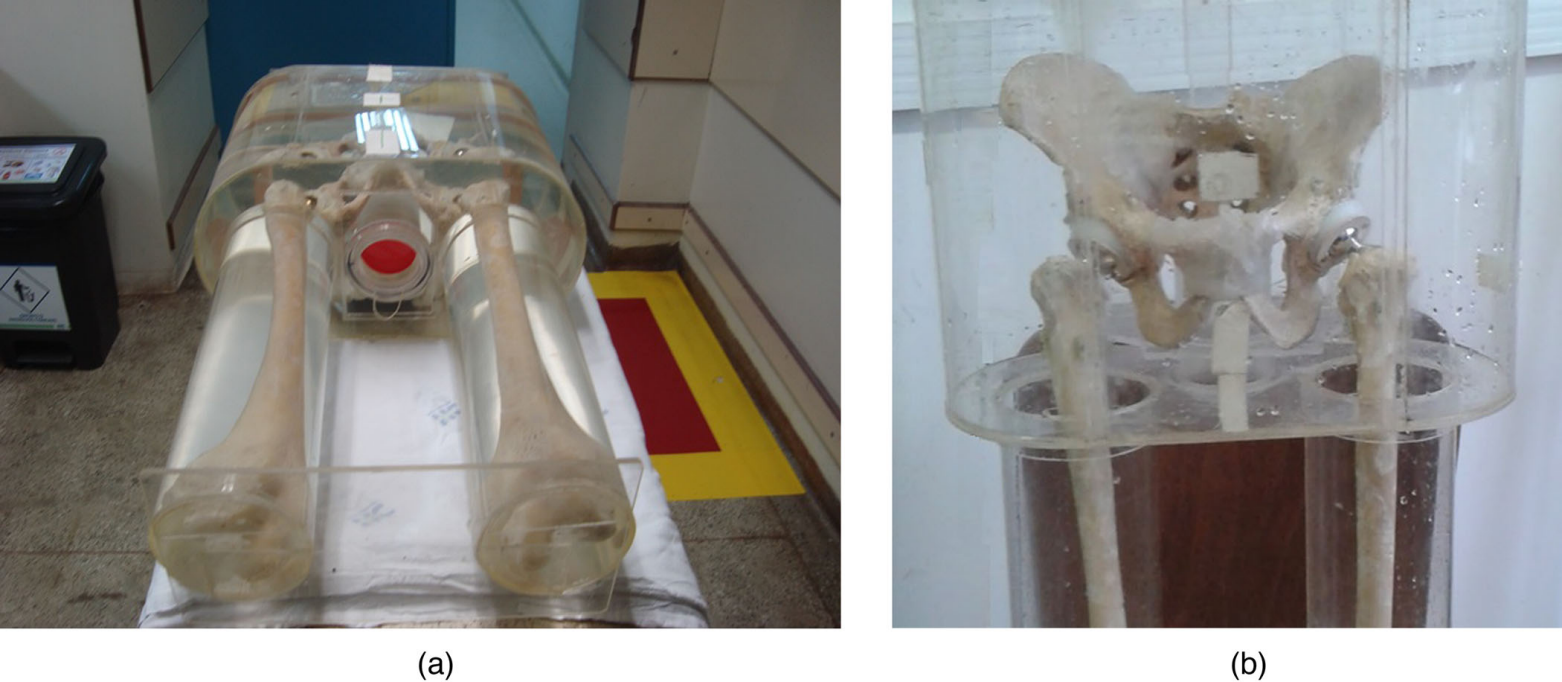
Pelvic phantom illustration. The central region shows the cavity with the container with MAGIC‐f polymer gel positioned (A). Anterior view of the pelvis details inside the phantom (B)

A pelvic region phantom in the real dimensions of an adult human with two femoral prostheses was used. It was constructed using an acrylic vessel and allowing insertion of the pelvis and the femoral prosthesis. The pelvis was composed of human bones where the polyethylene hip prostheses and the stainless‐steel femoral prosthesis were mounted. They were cemented using Baumer (Baumer.com.br) osseous methyl/polymethyl methacrylate cement and fixed using a radiopaque agent. An orthopedic surgeon installed the prostheses according to standard procedures. After positioning the bones, the phantom was filled with water to simulate soft tissue. The phantom also contained a cylindrical watertight cavity with 4.5 cm diameter and 5.0 cm length at the prostate region to accommodate the gel dosimeter vial with these dimensions, allowing to change the gel vial easily and quickly without having to open the phantom (Figure 1).

A 3D dose measurement of the target volume was done with the cylindrical vial filled with MAGIC‐f gel dosimeter and inserted in the phantom cavity. The TLDs were positioned at the femur, acetabulum, and sacrum for dose measurements.

### 2.2 TLD dosimetry

**FIGURE 2:**
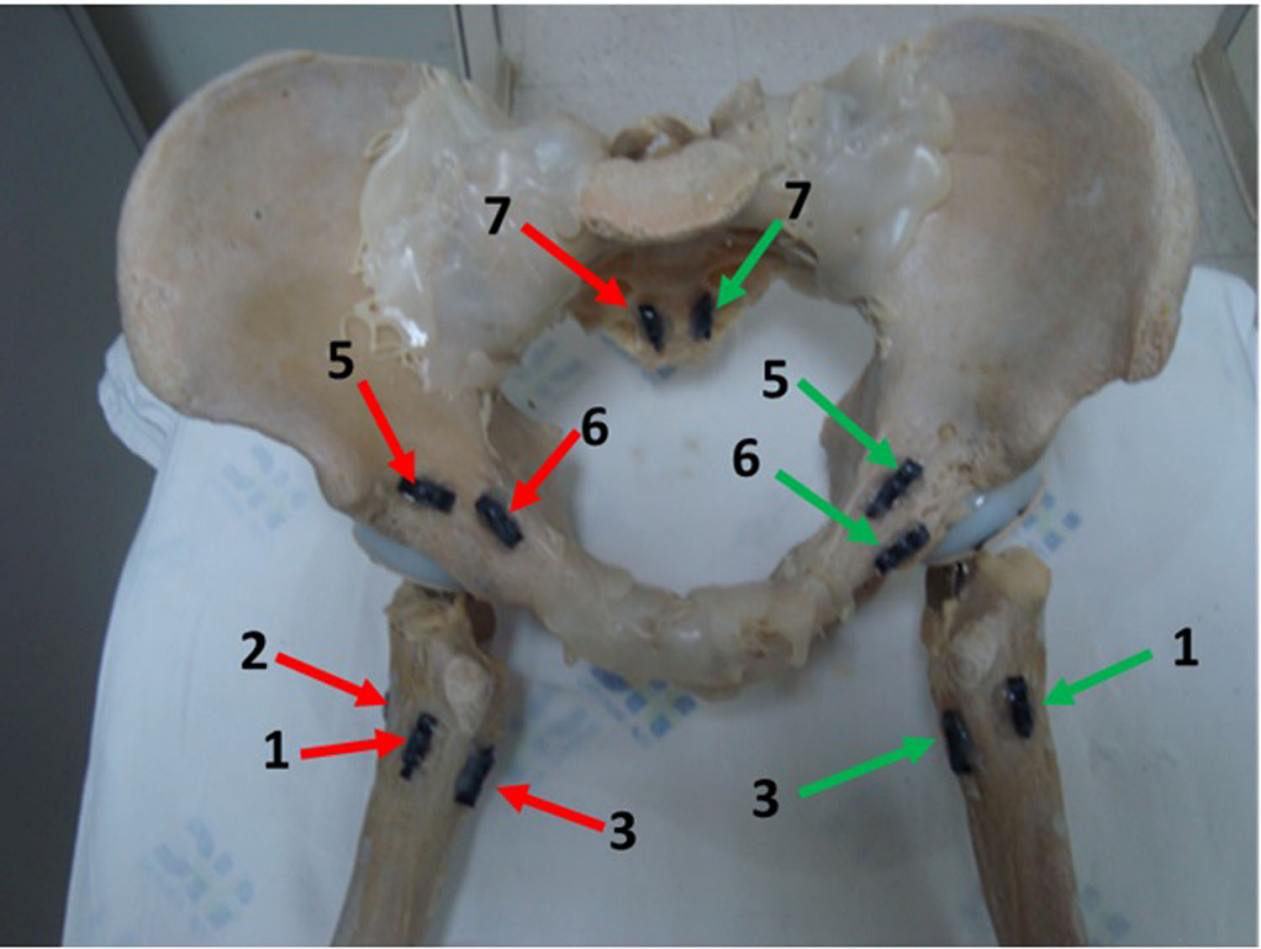
Anterior view of the TLD dosimeters distribution in the femur, acetabulum, and sacral bone. There were four dosimeters placed around the femur, two at the acetabulum, and one at the sacrum for each side. Red and green arrows represent the dosimeters on the right and left side of the pelvis, respectively

A total of 14 TLD dosimeters (LiF: Mg. Ti) were used; seven dosimeters were positioned on each side of the pelvis. They were glued on the pelvis after being encapsulated and sealed on a plastic tube. Figure 2 indicates their distribution on the phantom.

Dosimeters number 1‐4 were positioned around the femur, 5 and 6 at the acetabulum, and 7 at the sacrum for each side of the pelvis.

### 2.3 TLD preirradiation thermal treatment

A two‐stage thermal treatment before irradiation was applied to the TLD. The dosimeters were kept for 1 h in an oven at 400°C to restore their sensitivity. Next, they were placed in another oven for 2 h at 100°C to eliminate contributions of low‐temperature peaks to the signal reading.^19^

The TLDs were calibrated using a single beam irradiation in a fixed source–surface distance (SSD) of 100 cm. They were positioned at the depth of maximum dose in an acrylic phantom, and over 10 cm of acrylic plates to guarantee backscattering. A 20 × 20 cm^2^ irradiation field size was used and doses up to 10 Gy were delivered.

### 2.4 Gel dosimetry

**FIGURE 3:**
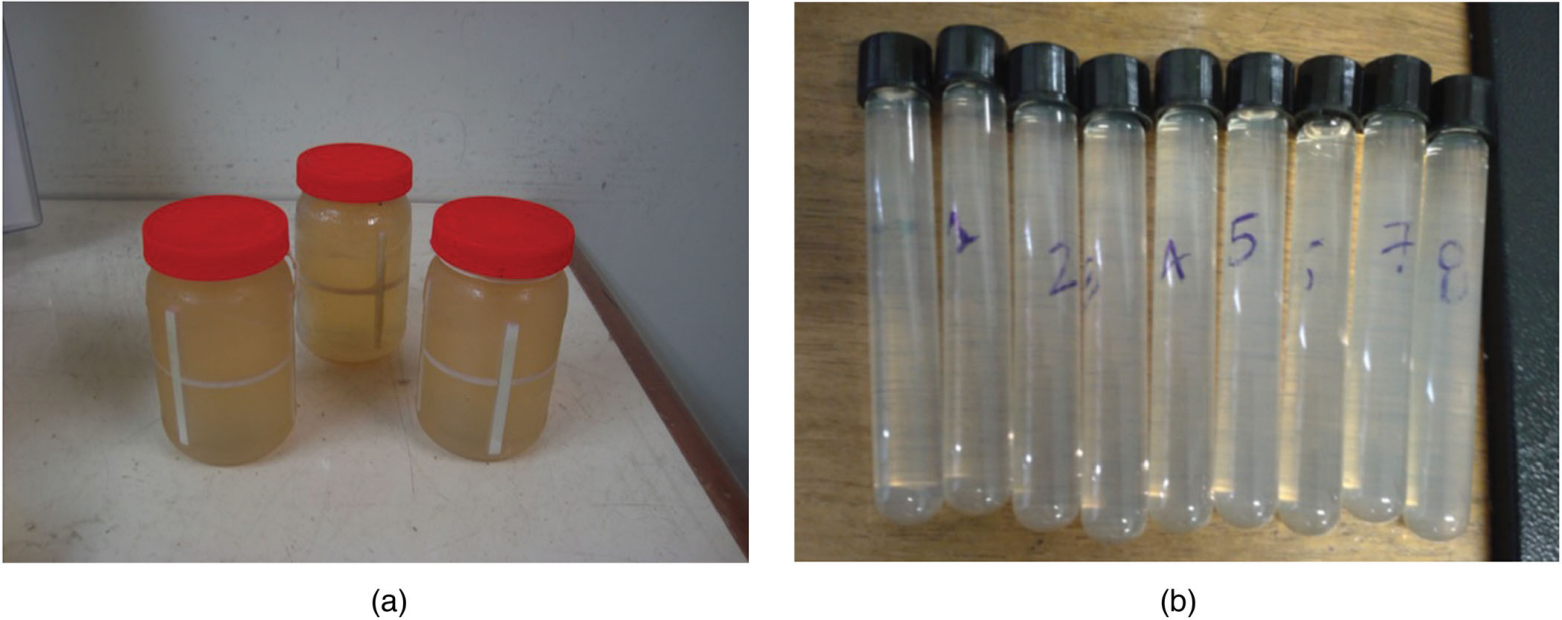
Airtight PET container used for the 3D dose measurement (A) and screwed cap sealed glass test tubes (9 mL) used as calibration vials (B)

MAGIC‐f gel dosimeter^14^, ^15^, ^16^, ^17^, ^18^ was used. It presents adequate dosimetric characteristics for use in 3D dosimetry in high‐energy beams and a high melting temperature, which makes its handling easier than other gel dosimeters.^14^, ^18^ It is composed of the following components and their respective mass concentrations: Mili‐Q Water (82.31%), bovine gelatin 300 Bloom (8.33%), ascorbic acid (0.03%), copper sulfate (0.02%), formaldehyde (3.32%), and methacrylic acid (5.99%). Gelatin was initially added to water at room temperature and mixed, then the solution was heated to 50°C, and this temperature was maintained until gelatin completely melted. After this step, the solution was cooled to 35°C; next, ascorbic acid, copper sulfate, and formaldehyde were added in this order. Methacrylic acid was then added after a few minutes, and the solution was stirred for 5 min more. The gel was poured in a cylindrical airtight plastic container (Figure 3A) made of polyethylene terephthalate (PET) for the 3D measurement. Eight screw‐capped test tubes (9 mL) were also filled and used for calibration purposes (Figure 3B). They were stored in a refrigerator at 7°C for at least 12 h before use.

The PET container fits precisely to the phantom aperture without any air gap. It was then removed from the phantom by pulling a 1 mm diameter strong cotton string. Plastic rods of different shapes (●, ■, ▲) were attached to the container inside the wall to work as fiducial marks and were used as orientation for the imaging registration process.

The gel dosimeter was also calibrated using a single beam, and the beam axis was parallel to the diameter of the cylindrical tubes. The calibration vials were positioned in an acrylic cast assuring positioning of the calibration vial's center at the maximum dose depth (1.5 cm), where the doses were calculated. This phantom was also placed over 10 cm of acrylic plates to ensure backscattering. The irradiation used a fixed SSD of 100 cm, a field size of 20 × 20 cm^2^ and doses up to 3 Gy were delivered.

### 2.5 Treatment planning and phantom irradiations

**FIGURE 4:**
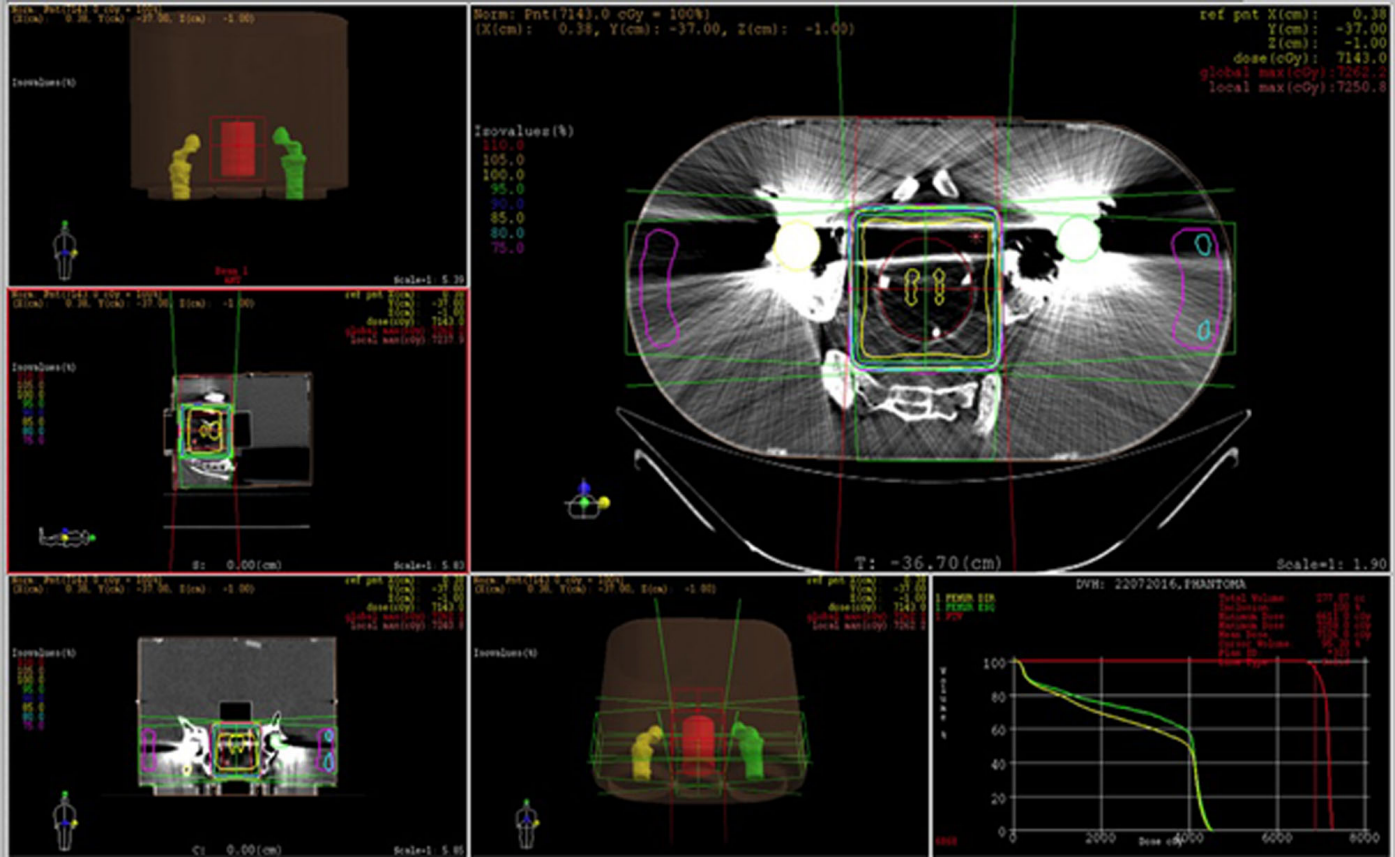
Screenshot of the TPS output for a bilateral prostheses prostate treatment using box technique irradiation

The treatment planning used the phantom CT images acquired with a Phillips Brilliance Big Bore scanner (Phillips). The PET gel dosimeter container volume was delineated as the planned target volume (PTV) in XIO TPS version 3.62 (Elekta, Stockholm AB, Sweden). A four‐field box plan using an isocentric setup to deliver 2 Gy to the PTV was created. Dose distributions were calculated without considering heterogeneity corrections due to the presence of artifacts from the higher atomic number of the prosthetic material. This option was used because we could, therefore, evaluate the effect of lacking correction in the TPS on the delivered dose. We were also aware of more recent and precise versions of TPS which were not available at our service. The treatment isocenter was positioned approximately at the center of the gel container, and the radiation fields encompassed the gel volume (*x* = 8.8 cm and *y* = 11.5 cm). The weight of each beam was adjusted until a homogeneous dose distribution was achieved (Figure 4).

The phantom was irradiated following the TPS plan and using a Siemens Primus (Siemens, Erlangen, Germany) linear accelerator (LINAC) after its alignment using the CT marks. Next, the TLDs and gel dosimeter calibration vials were irradiated following the setups described before and at the same LINAC.

### 2.6 TLD reading

The TLD reading used a Harshaw 2000 TLD reader. This equipment calibration followed the standard laboratory procedure before its use. The dosimeters used in this work were selected from a batch with its sensitivity being determined for the lot used in this experiment. The TLD dose values were compared to the mean dose value calculated by the TPS for the TLD volume delineated for each chip.

### 2.7 Gel dosimeter reading

MRI images of the gel container were acquired 24 h after irradiation with a Philips Achieva 3T scanner (Phillips). A multispin‐echo sequence with eight echoes equally spaced, multiples of 35 ms, and a repetition time of 1000 ms was used. The voxel size was 0.6 × 0.6 × 2.0 mm, and a matrix size of 224 × 224 × 60 was acquired. The phantom and the calibration vials were imaged immersed in a solution containing sodium chloride (NaCl) and manganese (II) chloride (MgCl_2_) to reduce the presence of susceptibility artifacts on images close to the phantom or vial edges. The gel vials were stored in the MRI room just after irradiation to complete the chemical reaction and thermal equilibration with the scanner room temperature.

Dose distributions were evaluated, employing R2 relaxometry in the MRI. All calculations used an in‐house developed software in MatLab (Mathworks Inc. Nattick, MA, USA). The R2 values for each calibration tube were evaluated by selecting a region of interest (ROI) at the center of the test tube, and avoiding the selection of the container glass wall to prevent errors. The mean R2 value and its standard deviation along all the calibration vials were calculated and related to the doses they received in the calibration curve.

**FIGURE 5:**
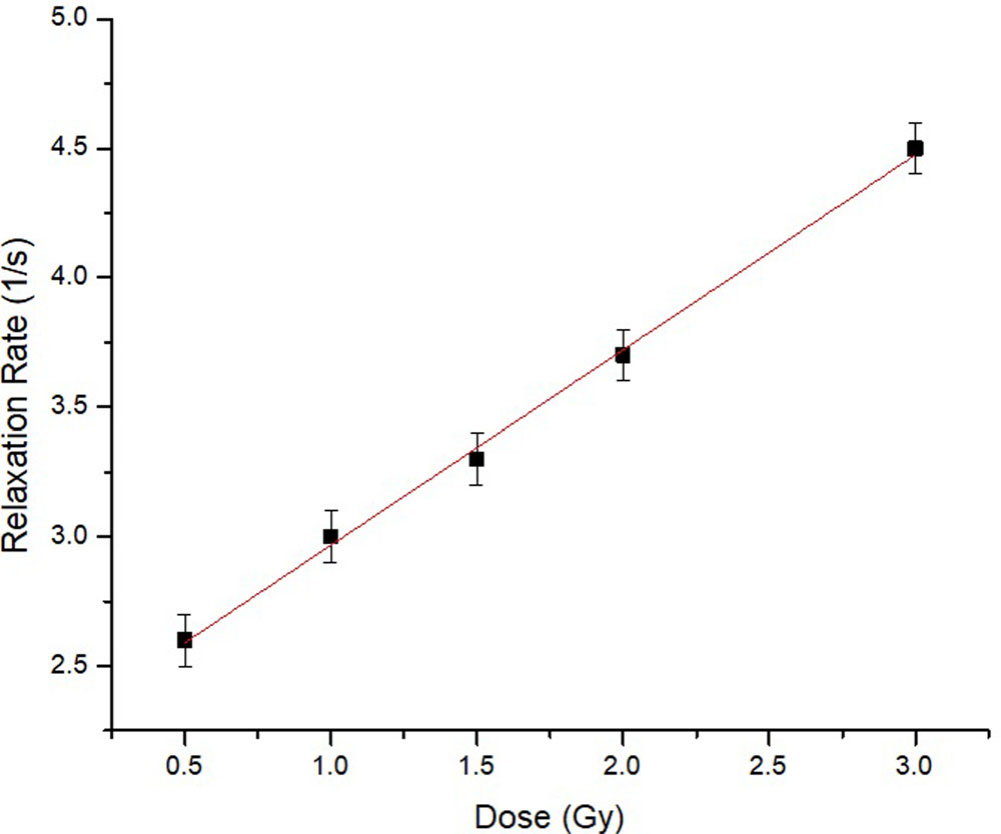
MAGIC‐f gel calibration curve showing a linear relationship: R2 = (2.21 ± 0.04) + (0.75 ± 0.02) D in the dose range measured, Pearson's *r *= 0.999

All the PET containers gel volume R2 values were also evaluated following the described methodology. We anticipated that the calibration curve would present a linear relation between R2 and dose (Figure 5 of section 3). Thus, the PET container R2 maps were normalized by the isocenter dose value and compared to the normalized TPS dose distribution using the 3D gamma evaluation with criteria of 3% of dose deviation, 3 mm distance to the agreement,^20^ and 15% threshold.

## 3 RESULTS

### 3.1 Magic‐f gel dosimetry results

The MAGIC‐f gel calibration curve shows a linear behavior between R2 relaxation rate and dose (Figure 5) with a sensitivity (slope of the calibration curve) value of 0.75 ± 0.02 Gy/s(*R* = 0.999). Error bars represent the standard deviations obtained from analyses of the same ROI on different slices of the gel vial irradiated with the corresponding dose.

**FIGURE 6:**
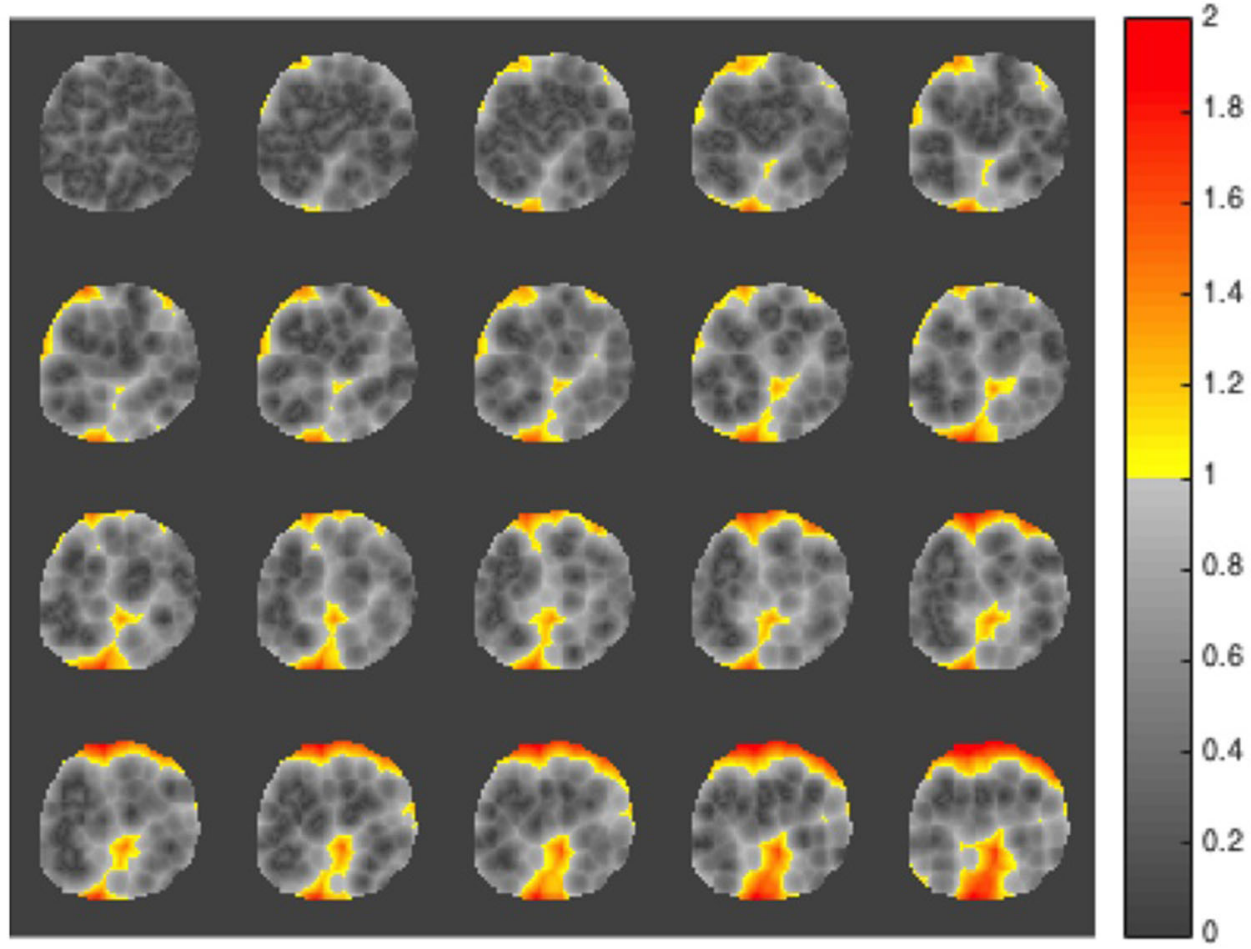
Examples of the axial gamma maps result in a comparison of the TPS dose distribution and the measured gel dose with 3%/3 mm criteria

The dose distributions measured by the gel and planned by the TPS were compared using 3D gamma analyses. The slice‐by‐slice results of the central region of the phantom are presented in Figure 6, resulting in a gamma passing rate of 93% of the points in a 3%/3 mm/15% threshold test.

### 3.2 Thermoluminescence results

**FIGURE 7:**
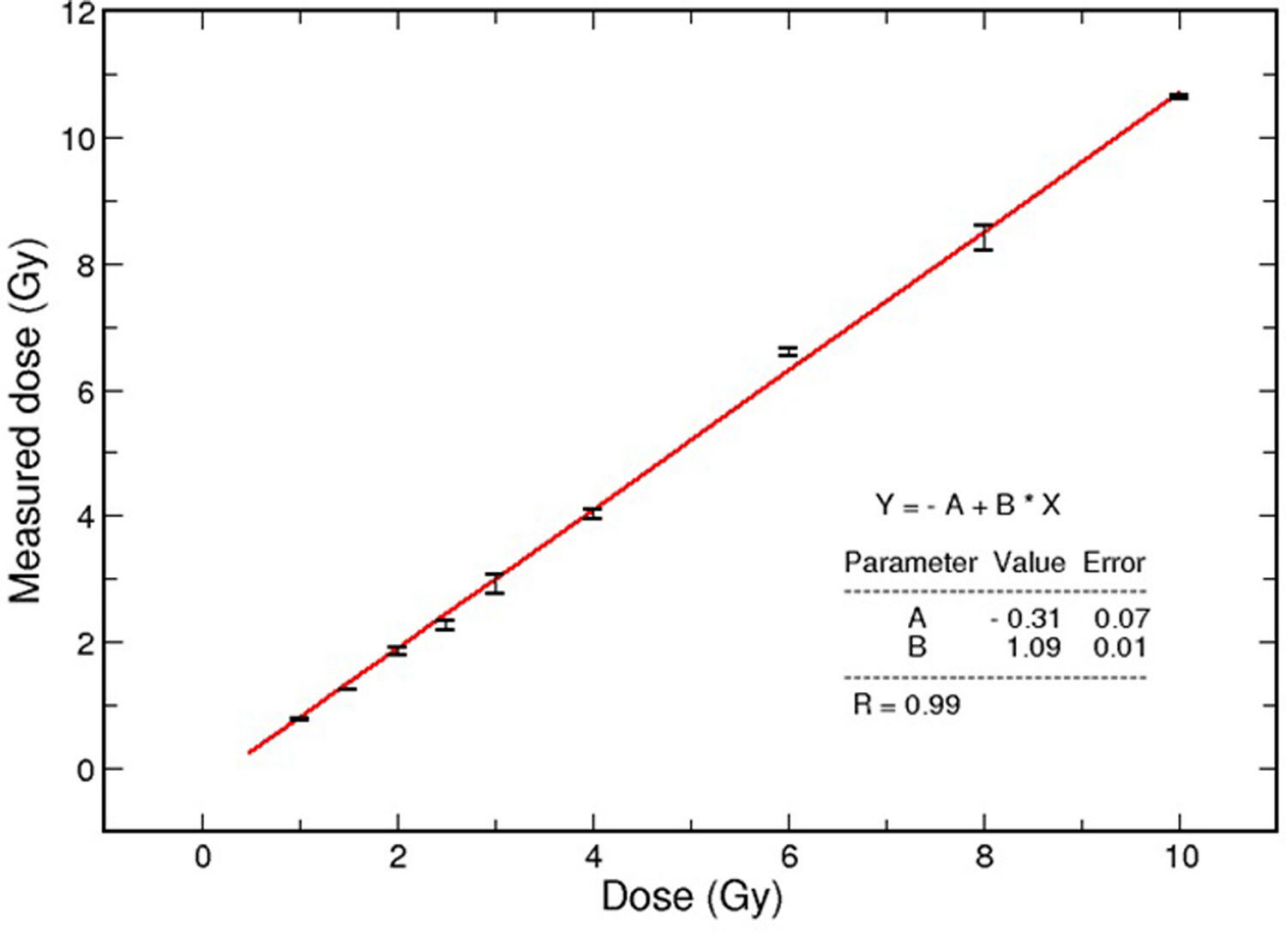
Correlation between the delivered doses and the measured dose for Li:Mg.Ti TL dosimeters

Figure 7 shows the correlation between the delivered dose and the measured dose for the TLD (*R* = 0.99). Table 1 presents the dose values measured for all the TLDs positioned in the phantom and their respective values calculated by the TPS.

**TABLE 1:** Dose measured with the thermoluminescent dosimeters and expected doses by the TPS calculation

		Left			Right		
Region	TLD number	TLD dose (Gy)	TPS dose (Gy)	Deviation (%)	TLD dose (Gy)	TPS dose (Gy)	Deviation (%)
Femur	1	0.5	0.6	−8.5	*	0.6	–
	2	1.4	1.2	12.0	0.8	0.5	55.6
	3	2.3	2.6	‐9.1	1.6	0.8	102.9
	4	2.2	2.2	0.0	1.5	0.6	150.0
Acetabulum	5	2.3	2.5	−7.2	2.6	2.6	−1.2
	6	3.9	3.8	1.3	2.3	2.5	−8.5
Sacrum	7	3.7	3.6	2.8	3.8	3.8	1.1

* The dose in this dosimeter was not determined because it made contact with water, compromising the measurement.

## 4 DISCUSSION

The gel dosimeter calibration curve was linear (Figure 5), thereby allowing comparison of the measured dose distributions to the expected dose distribution after normalization to their isocenter values. The gamma analysis results showed a passing rate of 93% of the points (Figure 6), indicating good agreement between the measured and calculated dose distributions. There are few fail points, mainly at the gel phantom's edges, attributed to MRI susceptibility artifacts^13^ and not specifically with dose discrepancies.

The correlation of the TLD measured doses with the delivered doses (Figure 7) in the calibration using a dose range up to 10 Gy (*R* = 0.99) assures that the dosimeters can be used in this study. However, the TLD measurements on the phantom present some disagreement between the measured dose and the TPS calculated values. These discrepancies are expected since our planning did not consider the heterogeneity corrections; furthermore, they reflect the metal's influence in the treatment dosimetry due to the radiation interaction with the high Z material of the prosthesis in the lateral beams.

TLD measurements at the femoral region with dosimeters 1 to 4 positioned on the left femur presented a mean variation of −1.4% to the TPS expected doses. Dosimeter 1 was positioned anteriorly to the femur, but it lay outside the treatment dose region, explaining its notable smaller dose than the others. In considering the low dose value evaluated, the variation of only 0.1 Gy (0.6‐0.5 Gy) to the TPS corresponds to −8.5%. Dosimeter 2 was positioned laterally to the femur in the outer part of the body; its reading was 12% higher than the planned dose. Although this is a significant difference, it could be due to the scattering of electrons from the stainless steel metal implant. In contrast, dosimeter 3, in the inner side of the femur, presented a reading which was 9.1% smaller than the planned reading, which reflects the effect of radiation attenuation in the steel alloy prosthesis. Finally, dosimeter 4, positioned in the posterior region of the femur, detected the same dose as planned.

The verified dose enhancement (+12%) is close to the 15% value found before using Monte Carlo simulations.^4^ Another study using a titanium prosthesis found dose enhancements between 21 and 30%.^5^ The observed dose reduction value (−9.1%) is also comparable to the variations achieved before, namely of 10‐45% using the titanium prosthesis^5^ and between 15 and 21% for the steel alloy^4^ prosthesis. Based on these data, it is evident that our results agree with the previous results reported. Still, a strict comparison of values depends on the prosthesis model since the prosthesis's thickness, composition, and geometry can alter its interaction with the radiation beam.

Dosimeters 1 to 4 of the right side presented higher deviations than their left‐side equivalent dosimeters. The critical variations found may be attributed to the treatment region since they were located at the border of the radiation treatment and close to the penumbral area, where smaller deviations in the positioning could significantly alter the results. The dosimeters in the CT acquisition used for treatment planning were positioned outside the treatment region, and they accordingly received a mean dose of 0.6 Gy. However, we hypothesized that this femur probably suffered a slight movement in the cranial direction during the phantom transportation or manipulations before the irradiation, which put the dosimeters inside the treatmentregion and resulted in a mean dose measurement of 1.3 Gy. It is worth noting that these dosimeters were positioned on the phantom before the treatment planning, which focused on the dose delivery in the prostate region containing the gel cylinder. It is also important to note that positioning is critical even for a realistic phantom, and movement of the parts can occur when moving the phantom through each treatment step. This movement is of even more concern when dealing with a patient.

The two dosimeters positioned on the acetabulum treatment region, one of each side of the pelvis, received high doses and presented deviations of approximately 1%. The other two dosimeters of this region (again one of each side of the pelvis) were displaced off their position, presenting deviations of approximately 8%. This result is also a critical spot due to the presence of different materials which lead to dose gradients, and small position changes may incur due to differences in the measured dose.

Dosimeters 7 were positioned on the sacral region of the pelvis, which corresponds to a stable area of the phantom and far from the metallic prosthesis, where they received the high doses of the treatment region. Therefore, their measurements were in excellent agreement with the planned measurements, with mean deviations smaller than 2%.

The dose discrepancies verified close to the prostheses have been used before to explain the prosthesis replacement in several patients. However, a study that compared the need for prosthesis replacement in women who had pelvic irradiation for gynecological cancer to women with breast cancer did not find any statistically significant higher risk of undergoing total hip replacement for the first group. The most common reason for a hip replacement in both groups was idiopathic osteoarthritis.^21^ However, dose discrepancies may be minimized if the AAPM TG‐63 guidelines are followed.^2^

Although the simple four‐field box technique was used in this study, it provided a homogeneous dose distribution, reducing the errors associated with the point‐dose measurements. The achieved results confirm the recommendation to avoid placing the treatment field directly on the hip prosthesis.^2^ This recommendation can be extrapolated to static beam IMRT because the radiation interaction with the metal may occur similarly. However, if the avoidance of beams crossing the metal is not respected, the uncertainty magnitude involved in the IMRT or VMAT deliveries may be smaller than in the 3D‐RT, because it depends on the dose delivered by each field or arc segments crossing the prosthesis, which may be smaller in the modulated deliveries.

The TLDs' results on the treatment region, which received the treatment doses (>3 Gy), are in agreement with the planned doses. The gel dosimetry 3D measurement at the treatment target volume also showed good agreement with the TPS plan. The dose divergences occurred in the vicinity of the treatment, at the prosthesis position, which receives approximately half of the treatment doses. The deviation values detected herein would probably be minimized with the use of heterogeneity correction. However, we preferred to evaluate the dosimetric deviations due to the metal presence in the beam and avoid heterogeneity correction uncertainties.^3^, ^22^ Among the circumvented uncertainties, we can include, first, inaccurate determination of the electron density of the prostheses material from the CT image following the Hounsfield unit (HU) to electron density correction curve.^23^, ^24^ To note, the curve used in this study was not extended to the highest HUs of the prosthesis materials. Second, difficulty in the delineation of the prosthesis region in the presence of CT image artifacts.^6^, ^25^ Third, miscalculations of the dose calculation algorithm version.^5^ Using a newer TPS algorithm would reduce the dose miscalculations. However, the other uncertainties would still be present in the study. Although this can be considered a limitation of the study, it can be important to indicate the absolute deviation values involved and help the clinical routine in planning these treatments.

Our study was designed based on a simple four‐field box technique without heterogeneity correction to reduce other uncertainties in the TPS calculation, affecting the agreement between calculated and measured values. The use of IMRT or VMAT could suffer additional undesirable variations due to small 3D dose variations (for example) which are not detected in planning QA procedures, or to the positioning of the TLD dosimeters in regions with high dose gradients. In addition, the TPS correction algorithms are in constant evolution to cope with its limitations, while having a good estimate of dose deviations without any correction is also useful.

## 5 CONCLUSIONS

In conclusion, our study presented a complete evaluation of the dose distribution at the simulated target volume and in the vicinity of the metallic implants of a simulated prostate treatment in a real anatomy phantom using a four‐field box technique. The dose measurements were compared to the TPS calculation without heterogeneity correction to identify the metal influence in the dose delivery. The 3D dose verification in the PTV region using gel dosimetry verified the agreement between the planned and delivered doses. In contrast, the TLD point dose measurements in the vicinity of the treatment showed important deviations in the delivered doses due to the use of irradiation beams crossing the metallic prosthesis.

## AUTHOR'S CONTRIBUTION

Conceptualization, investigation, and writing–original draft: Diana M. C. Rojas, Juliana F. Pavoni and Oswaldo Baffa. Resource, methodology, writing–review and editing: Diana M. C. Rojas, Juliana F. Pavoni, Gustavo V. Arruda, Oswaldo Baffa.

## CONFLICT OF INTEREST

The authors declare no conflicts of interest. This work is partially based on the Master's thesis of the first author which was coadvised by the coauthors.

## Supporting information

SUPPORTING INFORMATION

## Data Availability

The data that support the findings of this study are available from the corresponding author upon reasonable request.
