# Supplementary material for: Gel and thermoluminescence dosimetry for dose verifications of a real anatomy simulated prostate conformal radiation treatment in the presence of metallic femoral prosthesis
Source: J Appl Clin Med Phys. 2021 Aug 26;22(10):278–87. doi: 10.1002/acm2.13403 (PMC8504585; doi:10.1002/acm2.13403)
Supplement: Supplementary file 1 — SUPPORTING INFORMATION [file ACM2-22-278-s001.doc]

**Gel and Thermoluminescence dosimetry for dose verifications of a real anatomy simulated prostate conformal radiation treatment in the presence of metallic femoral prosthesis**

Diana M. C. Rojas, Ms.

Department of Physics

Faculty of Philosophy, Sciences and Letters at Ribeirão Preto – University of São Paulo

Av. Bandeirantes 3900, Monte Alegre, Ribeirão Preto, SP, Brazil - Zipcode: 14040-901

Juliana F. Pavoni, Ph.D.

Department of Physics

Faculty of Philosophy, Sciences and Letters at Ribeirão Preto – University of São Paulo

Av. Bandeirantes 3900, Monte Alegre, Ribeirão Preto, SP, Brazil - Zipcode: 14040-901

Gustavo V. Arruda, Ph.D.

Ribeirão Preto Medical School – University of São Paulo

Av. Bandeirantes 3900, Monte Alegre, Ribeirão Preto, SP, Brazil - Zipcode: 14040-900

Oswaldo Baffa, Ph.D.

Department of Physics

Faculty of Philosophy, Sciences and Letters at Ribeirão Preto – University of São Paulo

Av. Bandeirantes 3900, Monte Alegre, Ribeirão Preto, SP, Brazil - Zipcode: 14040-901

**Corresponding author:**

Prof. Juliana Fernandes Pavoni

Department of Physics, FFCLRP, USP

Av. Bandeirantes 3900, Monte Alegre, Ribeirão Preto, SP, Brazil

Zipcode: 14040-901

Phone: 55 16 3315.0082

Mobile: 55 16 99994.8666

Email: [jfpavoni@ffclrp.usp.br](mailto:jfpavoni@ffclrp.usp.br)

**Running title:**

Prostate radiotherapy in the presence of metallic femoral prosthesis

**Authorship statement**

- Conception and design of study: Juliana F. Pavoni, Oswaldo Baffa
- Acquisition of data: Diana M. C. Rojas, Juliana F. Pavoni, Oswaldo Baffa
- Analysis and/or interpretation of data: Diana M. C. Rojas, Juliana F. Pavoni, Gustavo V. Arruda, Oswaldo Baffa
- Drafting the manuscript: Diana M. C. Rojas
- Revising the manuscript critically for important intellectual content: Juliana F. Pavoni, Gustavo V. Arruda, Oswaldo Baffa
- Approval of the version of the manuscript to be published: Juliana F. Pavoni, Oswaldo Baffa

**Acknowledgements**

The technical support of L. Rocha. C.A. Brunello and C.R. Silva, and discussions with C.E. Garrido and A.C. Santos are greatly appreciated. Partial financial support: Grant 2021/02254-6, São Paulo Research Foundation (FAPESP). CEPID-Neuromat 13/07699-0. CNPq Grant 304107/2019-0, and CAPES finance code 001.
